# Slight dependence of temperate-forest herbaceous plants, *Geum urbanum* and *Senecio ovatus*, on arbuscular mycorrhizal fungi (AMF) enables them to colonise soils with a low level of AMF abundance

**DOI:** 10.1371/journal.pone.0258862

**Published:** 2021-10-26

**Authors:** Katarzyna Rożek, Kaja Rola, Szymon Zubek

**Affiliations:** Institute of Botany, Faculty of Biology, Jagiellonian University, Kraków, Poland; Free University of Bozen-Bolzano, ITALY

## Abstract

While numerous studies have revealed that arbuscular mycorrhizal fungi (AMF) enhance plant performance, the influence of these symbionts on temperate-forest herbaceous species in relation to soil physical and chemical properties has been left largely unexplored. Therefore, two perennial herbs, *Geum urbanum* (Rosaceae) and *Senecio ovatus* (Asteraceae), were examined in a laboratory pot experiment to determine whether AMF influenced their growth, photosynthetic performance index, and N and P contents in biomass. The treatments, involving three widespread AMF species, were prepared in the soils of two habitats colonised by both plants, namely beech and riparian forests, as follows: (1) control—soils without AMF, (2) *Claroideoglomus claroideum*, (3) *Funneliformis geosporus*, and (4) *Funneliformis mosseae*. Neither shoot mass nor photosynthetic performance index of *G*. *urbanum* and *S*. *ovatus* was enhanced by AMF. *Senecio ovatus* root mass was increased compared to control only by *F*. *geosporus*. Inconsistent effects were observed in N and P contents in shoots and roots of both species. The direction and magnitude of these responses were dependent on the fungal species and soil type. Although the plant species belong to families whose representatives are usually regularly colonised by and highly responsive to AMF, our study indicates that AMF had only a slight impact on the performance of *G*. *urbanum* and *S*. *ovatus* at the early stages of their development. The plants being slightly dependent on AMF are thus adapted to colonise temperate-forest soils with a low level of availability of AMF propagules.

## Introduction

Arbuscular mycorrhiza (AM) is one of the ubiquitous symbioses that frequently result in the enhancement of plant performance through nutritional and non-nutritional mechanisms [1]. However, the impact of arbuscular mycorrhizal fungi (AMF) on their partners is varied and may also be neutral or negative. It has been shown that AM enhances nutrient absorption and growth [2], competitiveness in communities [3, 4], and resistance to unfavourable environmental conditions [5–8] of certain species of plants. In other studies, AM had no effect, or a negative effect, on plant nutrition, growth, and diversity, because the cost of maintaining fungal partners exceeded the benefits resulting from this symbiosis [8–11]. In any case, in these relationships, environmental factors, physical and chemical properties of soil, and, most importantly, fungus and plant species identities were of significance [8, 12].

Previous studies on interactions between mycorrhizal fungi and plants in temperate forests focused overwhelmingly on ectomycorrhizas; however, the presence of endomycorrhizas, including AM, has been observed among forest overstorey and understorey plant species [13]. Studies focused on the impact of AMF on the herbaceous layer of forest ecosystems have shown that AMF are physically and functionally selective in terms of partners [14, 15], as well as being influenced by soil properties and plant diversity [16] and by habitat filtering processes [17]. Moreover, Moora et al. [18], using multispecies soil inocula, found that the influence of AMF on the growth, nutrient status, and AMF colonisation of understorey species is site-specific. Similarly, based on field observations of degrees of AMF root colonisation, it was suggested that AMF may play a role in supplying P to understorey species in sites characterised by the scant presence of this element [19]. Inconsistent effects of AMF soil inocula from young and old forest ecosystems on the growth of understorey plant species were found [20, 21]. On one hand, the were no differences between the effects of both AMF soil inocula on shoot and/or root mass [20]; on the other hand, they varied, with positive effects of AMF from old forests being linked with intensity of use and the impact thereof on AMF community composition [21]. However, heretofore no studies have focused on the impact of particular AMF species on the performance of herbaceous plants present in forests located in temperate climate zones in relation to soil physical and chemical properties. Therefore, two perennial herbs, *Geum urbanum* L. and *Senecio ovatus* (P. Gaertn., Mey. & Scherb.) Willd., were chosen in order to determine whether AMF influenced their performance. In laboratory conditions, we tested their response to inoculation with three widespread AMF species present in forest ecosystems around the world in soils of beech and riparian forests, which are colonised by both plant species.

The specific questions addressed in the present study are as follows: (1) Are the plants dependent on AMF for their performance? (2) To what extent do different AMF species affect plant mass, photosynthetic performance index, and N and P contents in biomass? (3) What is the relationship between degree of mycorrhizal colonisation and plant variables? (4) Are these interactions dependent on soils which differ in physical and chemical properties? Given functional diversity in AM symbioses, which is also affected by soil physical and chemical properties [8, 14], we expected that the effects of inoculation would differ between plant species, AMF species, and types of soil.

## Materials and methods

### Soils

Soils were collected from two localities in southern Poland: a beech forest adjacent to the Pazurek Nature Reserve near Olkusz (50°20ʹ40.3ʺ N, 19°38ʹ24.7ʺ E) and the riparian Łęgowski Forest in Cracow (50°03ʹ07.0ʺ N, 20°02ʹ03.8ʺ E). These habitats were selected because they are colonised by both *G*. *urbanum* and *S*. *ovatus* [19, 22]. The soils were collected to the depth of 20 cm, mixed and sieved, using mesh (2 cm). Three randomly chosen samples of each soil were passed through mesh (2 mm) and then analysed for physical and chemical properties, as specified in [23]. The properties of these soils are presented in Table 1. The soils were sterilised twice (121 °C; 2 h; one-week interval). Both localities are unprotected areas thus no permits for sampling of soils were required.

**Table 1:** Physical and chemical properties (mean ± SD; N = 3) of beech and riparian soils used in the experiment.

Soil properties	Soil type		
	beech	riparian	
Soil texture			
Sand (%)	67.00±0.00	22.33±0.58	*
Silt (%)	28.67±0.58	64.00±0.00	*
Clay (%)	4.33±0.58	13.67±0.58	*
pH	5.56±0.22	6.93±0.16	*
Total content			
C (%)	1.55±0.08	2.28±0.11	*
N (%)	0.13±0.01	0.24±0.00	*
P (mg kg^-1^)	139.50±21.80	252.00±7.15	*
K (mg kg^-1^)	900.11±60.86	6311.42±100.05	*
Mg (mg kg^-1^)	779.86±54.70	6023.45±206.01	*
Ca (mg kg^-1^)	1548.49±68.41	3399.90±86.16	*
Exchangeable content (mg kg^-1^)			
K	59.80±0.42	110.24±3.99	*
Mg	29.74±0.20	231.35±5.26	*
Ca	902.89±31.65	2954.69±80.18	*
N-NH_4_^+^	3.57±1.21	3.49±1.33	
N-NO_3_^-^	3.58±0.17	20.58±1.09	*
S-SO_4_	0.39±0.21	0.63±0.04	

Significant differences (p < 0.05) between soil types according to the Mann-Whitney U test are marked with asterisks.

### Fungi

Three globally widespread AMF species present in temperate forests in Europe [19, 24] were used: (1) *Claroideoglomus claroideum* (N.C. Schenck & G.S. Sm.) C. Walker & A. Schüßler [*Glomus claroideum* N.C. Schenck & G.S. Sm.], (2) *Funneliformis geosporus* (T.H. Nicolson & Gerd.) C. Walker & A. Schüßler [*Glomus geosporum* (T.H. Nicolson & Gerd.) C. Walker], and (3) *Funneliformis mosseae* (T.H. Nicolson & Gerd.) C. Walker & A. Schüßler [*Glomus mosseae* (T.H. Nicolson & Gerd.) Gerd. & Trappe]. Inocula of *C*. *claroideum*, *F*. *geosporus*, and *F*. *mosseae* were prepared in plastic pots (11 cm × 12 cm; 1400 ml in volume). Reference monoculture substrata (30 g) of BEG 23, BEG 11 and BEG 12 were softly mixed with sterile substratum (sand:expanded garden rock:rock phosphate; 3:1:50 g/L). Pots were planted with ca 50 seeds of *Plantago lanceolata* L. as a host plant [24]. The seeds were supplied by Herbador, Bydgoszcz, Poland. After 6 months, mixture of *P*. *lanceolata* roots (mycorrhizal frequency parameter was ca 50%), mycelia, and spores (ca 75 spores per 50 g), were used as AMF inocula. For the control treatment, *P*. *lanceolata* was grown in the same substratum without AMF [25]. No AMF mycelia were present in this material. The fungal species were derived from fungi collection of the Institute of Botany at the Jagiellonian University in Cracow. No permits for their use were required.

### Plants

Two herbaceous plant species were used in the experiment. *Geum urbanum* (wood avens) is a perennial herb which reproduces via rhizomes and seeds [26]. *Senecio ovatus* (wood ragwort) represents the same life form and reproduces via seeds [27]. These plants are present in several forest ecosystems [22], including beech and riparian forests of southern Poland. Seeds were collected in 2018 at two sites, 49°27ʹ20.6ʺ N, 20°43ʹ02.2ʺ E and 49°28ʹ26.2ʺ N, 20°43ʹ14.9ʺ E, in the Beskid Sądecki mountain range, near Piwniczna-Zdrój, for *G*. *urbanum* and *S*. *ovatus*, respectively, and stored in a refrigerator (2 months). Subsequently the seeds were surface-sterilised in 1% sodium hypochlorite solution for 15 minutes [28] and germinated in autoclaved sand in plastic pots (9 × 12.5 cm; 500 ml in volume) placed in closed Sun bags (Sigma-Aldrich), at 22 °C with 345 μmol PAR photons m^−2^ s^−1^, in a 12/12 h light regime. Both plants are unprotected species thus no permits for seed collections were required.

### Experimental setup

To the centre part of pots (9 × 12.5 cm; 500 ml in volume) with autoclaved soils (440 ml) of both types (Table 1) AMF inocula were added (30 g), as specified in Majewska et al. [25]. The controls were substratum (30 g) with non-mycorrhizal roots of *P*. *lanceolata*. Two seedlings (14 days old) of *G*. *urbanum* and *S*. *ovatus* were planted in the central part of each pot. After 14 days, from each pot, one individual was eliminated. The treatments in two soil types comprised (1) control, i.e. soil with no AMF, (2) *C*. *claroideum*, (3) *F*. *geosporus*, and (4) *F*. *mosseae*. In order to align bacterial compositions between the treatments, an aqueous filtrate of a triple inoculum mixture was added (4 ml; 20% suspension; w/v) as specified in Jansa et al. [29] and Majewska et al. [25]. For each treatment, we used eleven replicates, for a total of 176 pots (2 plant species × 2 types of soil × 4 treatments × 11 replicates). The pots were positioned randomly in the plant growth chamber and kept in closed Sun bags to avoid contamination (22 °C; 345 μmol PAR photons m^−2^ s^−1^; 12/12 h light regime). The plants were watered once a week by 50 ml of sterile water per pot.

### Plant harvesting

After 75 days experiment was finished to estimate plant performance at the early stages of development. In order to evaluate photosynthetic parameters, chlorophyll *a* fluorescence were measured. Following this procedure, all plants were harvested, cleansed of soil particles in tap water, and washed twice in distilled water. Each specimen was divided into shoots and roots. One-fifth of the roots of each individual plant was cut and stained in order to observe AMF structures and to calculate degree of colonisation. The shoots and the remainder of the roots were dried at 22 °C and weighed with an analytical balance (Radwag, WPA 60/c/1; 0.0001 g precision level) in order to evaluate mass. The shoots and roots were also used for measurements of nitrogen (N) and phosphorus (P) contents.

### Chlorophyll *a* fluorescence measurements

Chlorophyll *a* fluorescence was measured by a fluorimeter Handy PEA (Hansatech Instruments Ltd., King’s Lynn, Norfolk, UK) in accordance with Strasser et al. [30] and Tsimilli-Michael and Strasser [31]. From each pot three intact and mature leaves of *G*. *urbanum* and *S*. *ovatus* were dark-adapted for 30 min prior measurements For each sample average OJIP fluorescence transients were calculated in accordance with the JIP test [30] with Biolyzer software (Laboratory of Bioenergetics, University of Geneva, Switzerland). The performance index (PI_ABS_), which estimate the overall photosynthetic performance [31], was presented.

### Determination of degree of AMF root colonisation

Roots were stained in accordance with the method of Phillips and Hayman [32], with modifications [33]. Thirty randomly selected fragments (1 cm) of fine roots were placed on slides in glycerol:lactic acid (1:1; v/v) and squashed using cover slides. The chemicals used, of pure quality for analysis, were produced by Chempur, Poland. AMF colonisation was calculated in accordance with the method of Trouvelot et al. [34], using a Nikon Eclipse 80i light microscope with Nomarski interference contrast. The analysed parameters comprised mycorrhizal frequency (F), relative mycorrhizal root length (M), and relative arbuscular richness (A) [34].

### Measurement of nitrogen and phosphorus contents in shoots and roots

Shoots and roots of *G*. *urbanum* and *S*. *ovatus* were dried at 80 °C and then milled with a Pulverisette 14 variable-speed rotor mill (Fritsch, Germany). The contents of N (%) in shoots and roots were measured using the Kjeldahl method. Samples were digested in H_2_SO_4_ with Kjeltabs (K_2_SO_4_ + CuSO_4_ · 5H_2_O; Foss Tecator Digestor Auto) followed by distillation on a Foss Tecator Kjeltec 2300 Digestion Analyzer Unit (Foss Tecator; AN 300 v. 4.0). Contents of P (mg kg^–1^) in shoots and roots were measured colourimetrically with a Hach Lange DR 3800 using the vanadate-molybdate method [35]. The samples were digested in a hot concentrated mixture of HNO_3_ and HClO_4_ (4:1; Foss Tecator Digestor Auto). These suprapure-grade nitric (69–70%) and perchloric (70–72%) acids were produced by J.T. Baker and Merck, respectively. Analytical grade sulphuric acid (95–97%) was produced by J.T. Baker. In the case of *S*. *ovatus*, due to an insufficient mass of roots, only P contents were measured.

### Calculations and statistical analyses

The mycorrhizal dependency (Md) of *G*. *urbanum* and *S*. *ovatus*, an indicator of the degree of dependence of plants on AMF for maximum growth, was calculated according to the equation: Md = [1 − (mean total biomass of plants without AMF/ total biomass of each plant inoculated with AMF)] × 100% [36, 37]. Similarly, we used the Md parameter in conjunction with the contents of N and P in biomass (shoots and roots) to calculate the degree of dependence of plants on AMF for acquisition of nutrients: Md = [1 − (mean total contents of N or P in biomass of plants without AMF / total contents of N or P in biomass of each plant inoculated with AMF)] × 100%.

A non-parametric Mann-Whitney U test was performed to test the significance of differences in soil physical and chemical properties between the two soil types.

Two-way analysis of variance, followed by Tukey’s (HSD) test, with fungal species and soil type as categorical predictors was performed to test the differences in mycorrhizal parameters (F, M, and A) across AMF-inoculated treatments, as well as mass of shoots and roots, PI_ABS_, N and P contents in shoots and roots, mycorrhizal dependency for total biomass (shoots and roots), and N and P contents in total biomass across all treatments, for *G*. *urbanum* and *S*. *ovatus*, separately. Prior to the analysis, the assumption of normality was verified using the Lilliefors corrected Kolmogorov-Smirnov test. Levene’s test was used to assess homogeneity of variance.

Since three mycorrhizal parameters were strongly correlated with each other (Pearson’s R > 0.9), only relative mycorrhizal root length (M) was included in further analyses. The relationships between the M parameter and mass of shoots and roots, the PI_ABS_ parameter, and N/P contents in shoots and roots were tested with Pearson’s correlation coefficients, separately for *G*. *urbanum* and *S*. *ovatus*, AMF inocula, and soil types.

Plant parameters (M, root and shoot mass, PI_ABS_, N and P contents in shoots and roots) were also investigated with principal component analysis (PCA) to identify the relationships among these parameters and to recognize whether samples are grouped according to different soil types and fungal treatments. The analysis was based on the correlation matrix and performed separately for *G*. *urbanum* and *S*. *ovatus*.

All treatments involved 11 replicates. Two replicates of *S*. *ovatus* planted on riparian soil and inoculated with *F*. *geosporus* were excluded from the analysis. Due to the lack of data for the N content in *S*. *ovatus* roots, analyses concerning this parameter and the mycorrhizal dependency for the total N content in the biomass for *S*. *ovatus* was not performed. In the case of single missing data for the N content in *G*. *urbanum* roots and *S*. *ovatus* shoots, Tukey’s (HSD) test for unequal sample sizes was applied. The analyses were performed using STATISTICA 10 (StatSoft, Tulsa, Oklahoma, USA) and PAST 3.22 [38].

## Results

### AMF root colonisation

**Fig 1:**
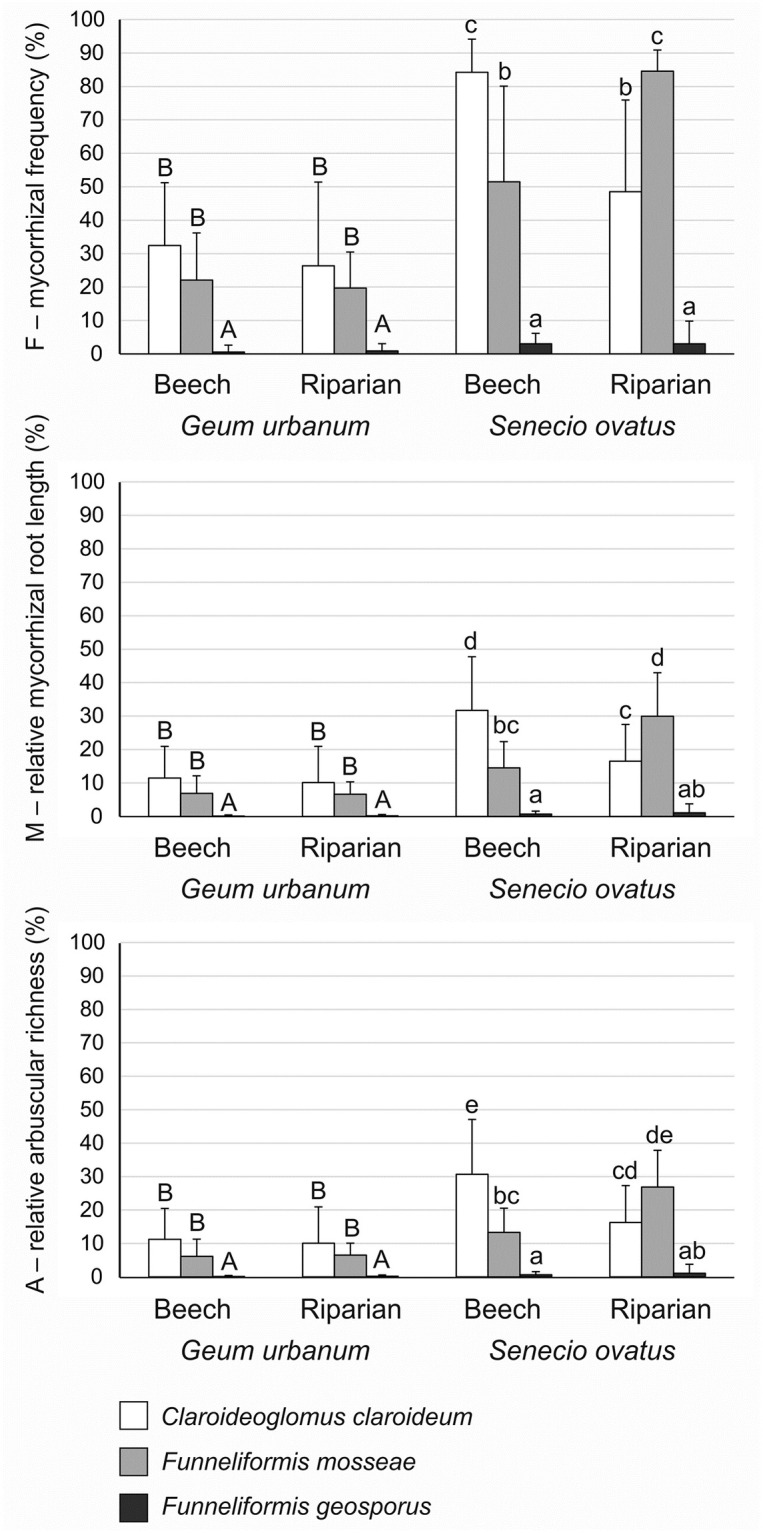
Mycorrhizal colonisation (percentages; mean ± SD) of *Geum urbanum* and *Senecio ovatus* grown in beech and riparian soils. Mycorrhizal parameters: mycorrhizal frequency (F); relative mycorrhizal root length (M); relative arbuscular richness (A). Within each plant species, lowercase letters above the bars indicate statistically significant interaction between fungus and soil effects; uppercase letters indicate the significant main effect of fungus; different letters above the bars indicate statistically significant differences; for each, p < 0.05. See Table 2 for details on the main effects and interactions.

In general, the level of AMF colonisation of *G*. *urbanum*, represented by the three mycorrhizal parameters (mycorrhizal frequency—F, relative mycorrhizal root length—M, and relative arbuscular richness—A), was low in the case of all treatments. Higher levels of mycorrhizal colonisation were found in *S*. *ovatus* (Fig 1). Two-way ANOVA revealed that, in both soils, the F, M, and A parameters of *G*. *urbanum* were higher in *C*. *claroideum* and *F*. *mosseae* treatments than in *F*. *geosporus* (significant fungus effect; Table 2). In contrast, significant fungus × soil effects were found for all mycorrhizal parameters in *S*. *ovatus* (Table 2). The highest level of colonisation in plants in beech soil treatments was found for *C*. *claroideum*, in riparian soil treatments for *F*. *mosseae*. The level of colonisation of *F*. *geosporus* was the lowest in both soils (Fig 1).

**Table 2:** Results of two-way ANOVA for the effects of fungal species, soil types, and their interaction on *Geum urbanum* and *Senecio ovatus* parameters; the effects in bold are statistically significant (p < 0.05).

Plant parameters		*Geum urbanum*										*Senecio ovatus*									
		Fungus			Soil			Fungus × Soil			Error	Fungus			Soil			Fungus × Soil			Error
		F	p	df	F	p	df	F	p	df	df	F	p	df	F	p	df	F	p	df	df
Mycorrhizal parameters	F—mycorrhizal frequency (%)	**21.87**	**<0.001**	**2**	0.57	0.455	1	0.26	0.774	2	60	**92.97**	**<0.001**	**2**	0.04	0.837	1	**21.51**	**<0.001**	**2**	58
	M—relative mycorrhizal root length	**15.42**	**<0.001**	**2**	0.11	0.742	1	0.08	0.925	2	60	**32.17**	**<0.001**	**2**	0.01	0.922	1	**12.17**	**<0.001**	**2**	58
	A—relative arbuscular richness	**15.18**	**<0.001**	**2**	0.02	0.890	1	0.09	0.918	2	60	**30.89**	**<0.001**	**2**	0.00	0.965	1	**10.90**	**<0.001**	**2**	58
Shoot mass (g)		0.30	0.823	3	0.43	0.516	1	1.79	0.155	3	80	1.59	0.199	3	0.00	0.951	1	1.87	0.141	3	78
Root mass (g)		**5.03**	**0.003**	**3**	**30.09**	**<0.001**	**1**	0.30	0.823	3	80	**5.94**	**0.001**	**3**	**5.77**	**0.019**	**1**	1.76	0.161	3	78
PI_ABS_—photosynthetic performance index		2.07	0.111	3	0.05	0.831	1	1.04	0.379	3	80	**3.87**	**0.012**	**3**	**17.83**	**<0.001**	**1**	**3.25**	**0.026**	**3**	78
Nitrogen content in shoots (%)		1.50	0.220	3	**34.02**	**<0.001**	**1**	1.48	0.225	3	80	2.04	0.116	3	**12.77**	**0.001**	**1**	**3.95**	**0.012**	**3**	72
Nitrogen content in roots (%)		**22.39**	**<0.001**	**3**	**27.25**	**<0.001**	**1**	**8.59**	**<0.001**	**3**	74										
Phosphorus content in shoots (mg kg^-1^)		**9.52**	**<0.001**	**3**	**8.25**	**0.005**	**1**	0.15	0.931	3	80	1.64	0.186	3	**6.07**	**0.016**	**1**	**3.42**	**0.021**	**3**	78
Phosphorus content in roots (mg kg^-1^)		**18.60**	**<0.001**	**3**	**40.72**	**<0.001**	**1**	**4.41**	**0.006**	**3**	80	**11.99**	**<0.001**	**3**	**34.10**	**<0.001**	**1**	**4.00**	**0.011**	**3**	78
Mycorrhizal dependency—biomass (%)		0.11	0.898	2	0.05	0.831	1	1.29	0.283	2	60	2.60	0.083	2	0.09	0.769	1	1.93	0.154	2	58
Mycorrhizal dependency—N in biomass (%)		**10.06**	**<0.001**	**2**	0.01	0.946	1	**6.21**	**0.004**	**2**	57										
Mycorrhizal dependency—P in biomass (%)		**22.85**	**<0.001**	**2**	**14.10**	**<0.001**	**1**	2.67	0.078	2	60	**15.39**	**<0.001**	**2**	**43.24**	**<0.001**	**1**	0.09	0.913	2	58

No measurements of nitrogen content in roots of *S*. *ovatus* were conducted due to the small mass of roots.

### Plant growth

**Fig 2:**
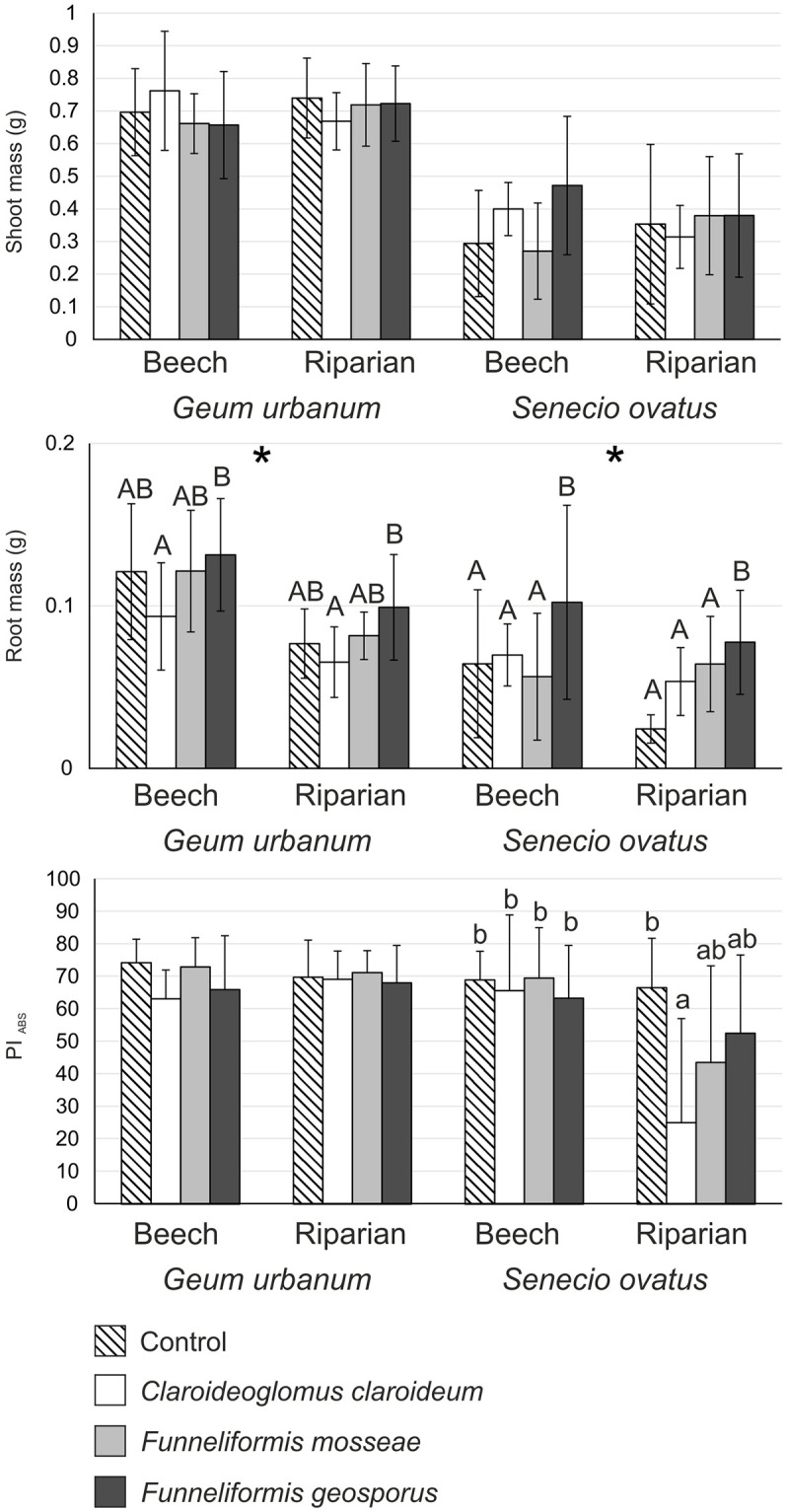
Shoot and root dry mass (grams; mean ± SD) and photosynthetic performance index (PI_ABS_; mean ± SD) of *Geum urbanum* and *Senecio ovatus* grown in beech and riparian soils. Within each plant species, lowercase letters above the bars indicate statistically significant interaction between fungus and soil effects; uppercase letters indicate the significant main effect of fungus; different letters above the bars indicate statistically significant differences; the asterisk (*) indicates the significant main effect of soil; for each, p < 0.05. See Table 2 for details on the main effects and interactions.

Neither fungus nor soil had a significant effect on the shoot mass of both plant species. On the other hand, we found that both factors had a significant effect on the mass of roots (significant fungus and soil effects; Table 2). The mass of roots was on average 40.6% higher in beech soil treatments compared to riparian soil treatments (significant soil effect). As regards fungus effect, *F*. *geosporus* treatments were characterised by a significantly greater mass of *G*. *urbanum* roots than *C*. *claroideum* treatments for both soil types. In the case of *S*. *ovatus*, *F*. *geosporus* treatments were characterised by a significantly greater root mass compared to the remaining treatments (Fig 2).

### Photosynthetic performance index

For *G*. *urbanum*, no significant effects were found. The value of PI_ABS_ of *S*. *ovatus* was significantly lower only in *C*. *claroideum* treatment in riparian soil compared to control treatment in riparian soil and all treatments in beech soil (Table 2; Fig 2).

### Nitrogen and phosphorus contents in shoots and roots

**Fig 3:**
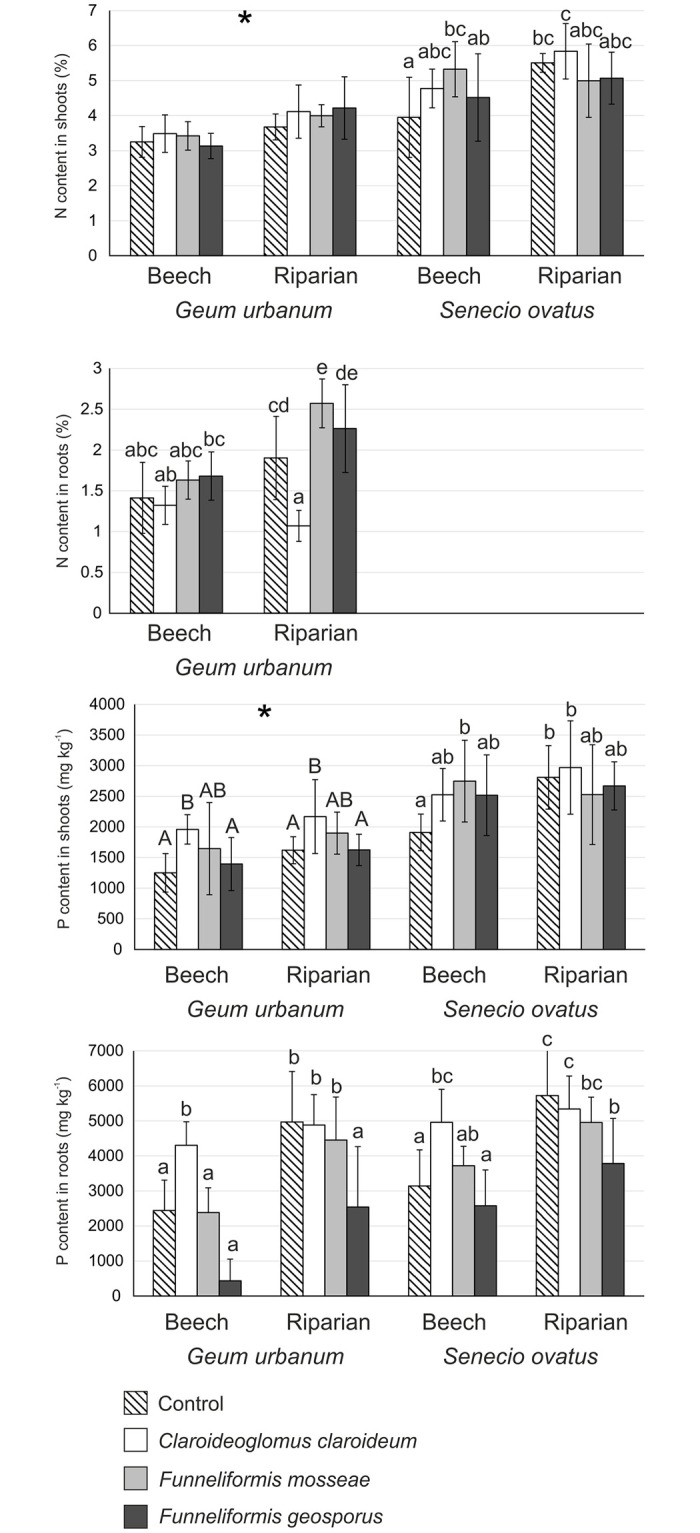
Contents of nitrogen and phosphorus (milligrams per kilogram dry weight; mean ± SD) in the shoots and roots of *Geum urbanum* and/or *Senecio ovatus* grown in beech and riparian soils. Within each plant species, lowercase letters above the bars indicate statistically significant interaction between fungus and soil effects; uppercase letters indicate the significant main effect of fungus; different letters above the bars indicate statistically significant differences; the asterisk (*) indicates the significant main effect of soil; for each, p < 0.05. See Table 2 for details on the main effects and interactions.

N contents in shoots of *G*. *urbanum* differed significantly only between soil types (significant soil effect; Table 2), being higher on riparian soil. For *S*. *ovatus*, significant fungus × soil interaction was revealed (Table 2). Control treatment in beech soil was characterised by significantly lower N content in shoots compared to *F*. *mosseae* in beech soil and *C*. *claroideum* and control treatment in riparian soil (Fig 3).

N content in roots of *G*. *urbanum* was influenced by both fungal species and soil type (significant fungus × soil interaction; Table 2). *F*. *mosseae* was most effective in the enhancement of N root content in riparian soil (Fig 3).

Significant effects of both fungus and soil on P contents were noted in shoots of *G*. *urbanum* (Table 2). In the case of both soil types, *C*. *claroideum* was most effective in the enhancement of P shoot contents. Nevertheless, lower values of this parameter were recorded in shoots of plants in beech soil in comparison with riparian soil (Fig 3). As regards *S*. *ovatus*, two-way ANOVA revealed significant fungus × soil interaction (Table 2). Plants from the control treatment and inoculated with *C*. *claroideum* in riparian soil as well as those inoculated with *F*. *mosseae* and planted in beech soil showed increased contents of P in shoots compared to control treatment in beech soil (Fig 3).

Significant fungus × soil interaction in P contents in roots was noted for both plant species (Table 2). *C*. *claroideum* was most effective in the enhancement of contents of this element in beech soil, differing significantly from all other treatments for *G*. *urbanum* as well as from control and *F*. *geosporus* for *S*. *ovatus*. In riparian soil, higher contents of P in roots of *G*. *urbanum* were recorded for control, *C*. *claroideum*, and *F*. *mosseae* than for *F*. *geosporus*. In the case of *S*. *ovatus*, control and *C*. *claroideum* treatments differed from *F*. *geosporus* (Fig 3).

### Mycorrhizal dependency in relation to plant growth and N and P acquisition

**Fig 4:**
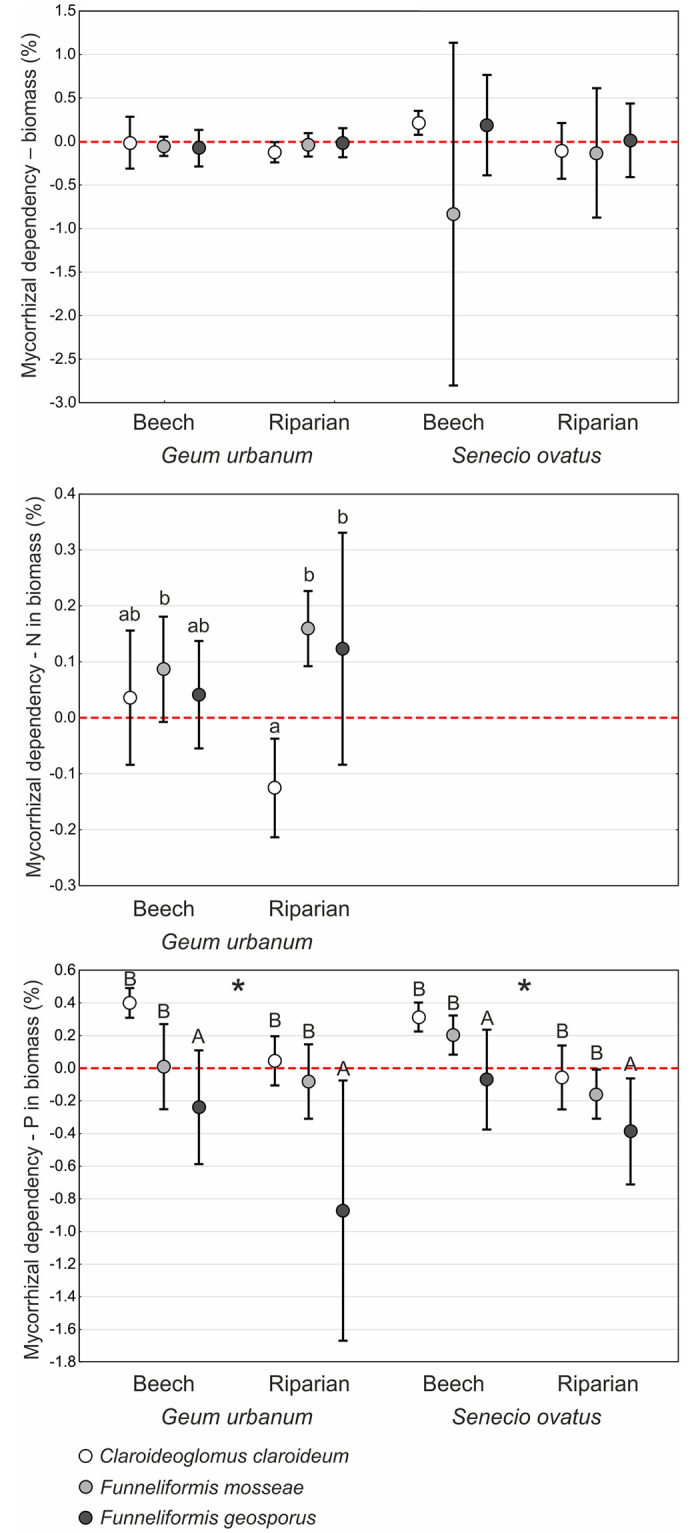
Mycorrhizal dependency parameters (percentages; mean ± SD) of values of mass of shoots and roots as well as of nitrogen and phosphorus contents in shoots and roots of *Geum urbanum* and/or *Senecio ovatus* grown in beech and riparian soils. Within each plant species, lowercase letters above the bars indicate statistically significant interaction between fungus and soil effects; uppercase letters indicate the significant main effect of fungus; different letters above the bars indicate statistically significant differences; the asterisk (*) indicates the significant main effect of soil; for each, p < 0.05. See Table 2 for details on the main effects and interactions.

The mycorrhizal dependency of *G*. *urbanum* and *S*. *ovatus* in relation to biomass was very low, rarely exceeding zero (Fig 4). The results of two-way ANOVA indicated no significant effects of fungus or soil in the case of either plant species (Table 2).

Mycorrhizal dependency related to N and P contents in biomass of *G*. *urbanum* and *S*. *ovatus* was also low. Their values did not exceed 0.5, and, in the cases of several treatments, were less than zero (Fig 4).

For *G*. *urbanum*, mycorrhizal dependency related to N contents in biomass were significantly lower in *C*. *claroideum* in riparian soil than in *F*. *geosporus* in riparian soil and *F*. *mosseae* in both soil types (significant fungus × soil interaction; Table 2, Fig 4).

Both fungus and soil had a significant effect on mycorrhizal dependency related to P contents in biomass in both plant species (Table 2). Treatments of *C*. *claroideum* and *F*. *mosseae* showed significantly higher values than those inoculated with *F*. *geosporus* in both soil types and plant species. Significantly lower values were recorded for riparian soil compared to beech soil treatments (Fig 4).

### Relationships between degree of AMF colonisation, shoot and root mass, photosynthetic performance index, and contents of N and P

The relationship of mycorrhizal colonisation intensity, expressed as the relative mycorrhizal root length parameter (M), with shoot and root mass and N and P contents differed between AMF species and soil types, although in only a few parameters. A significant positive correlation was found between M values and N content in the shoots of *G*. *urbanum* inoculated with *C*. *claroideum* in riparian soil. *F*. *geosporus* significantly increased N content in *G*. *urbanum* roots, but also significantly reduced P contents in shoots; this effect was observed only in riparian soil. As regards *S*. *ovatus*, significant correlations were recorded only for *F*. *mosseae*. P contents in roots increased along with increasing mycorrhizal colonisation of this species in beech soil treatments. The same effect was observed for P contents in shoots in riparian soil treatments (Table 3).

**Table 3:** Pearson’s correlation coefficients between relative mycorrhizal root length and mass of shoots and roots, photosynthetic performance index, and contents of phosphorus and nitrogen in *Geum urbanum* and *Senecio ovatus* for particular arbuscular mycorrhizal fungi species treatments and soil types.

Plant parameters	*Geum urbanum*						*Senecio ovatus*					
	*Claroideoglomus claroideum*		*Funneliformis mosseae*		*Funneliformis geosporus*		*Claroideoglomus claroideum*		*Funneliformis mosseae*		*Funneliformis geosporus*	
	Beech	Riparian	Beech	Riparian	Beech	Riparian	Beech	Riparian	Beech	Riparian	Beech	Riparian
Shoot mass (g)	0.00	0.23	0.19	0.26	0.26	0.54	-0.02	-0.04	0.45	-0.51	0.37	-0.28
Root mass (g)	-0.32	-0.48	0.15	-0.24	-0.17	-0.38	-0.29	-0.50	0.26	-0.31	-0.18	-0.08
PI_ABS_—photosynthetic performance index	-0.02	0.27	-0.27	0.30	-0.12	0.42	0.37	-0.11	-0.07	-0.25	-0.06	0.04
Nitrogen content in shoots (%)	-0.07	**0.73**	0.25	0.24	0.23	0.44	-0.59	-0.31	0.12	-0.29	-0.15	0.55
Nitrogen content in roots (%)	0.07	-0.04	-0.33	0.29	-0.03	**0.66**						
Phosphorus content in shoots (mg kg^-1^)	0.36	0.51	-0.38	0.55	-0.31	**-0.63**	0.44	-0.21	-0.19	**0.64**	0.00	0.50
Phosphorus content in roots (mg kg^-1^)	0.42	0.20	-0.21	0.57	-0.13	-0.07	-0.05	0.06	**0.60**	-0.48	-0.16	-0.44

Significant correlations (p < 0.05) are shown in bold. No measurements of nitrogen content in roots of *S*. *ovatus* were conducted due to the small mass of roots.

**Fig 5:**
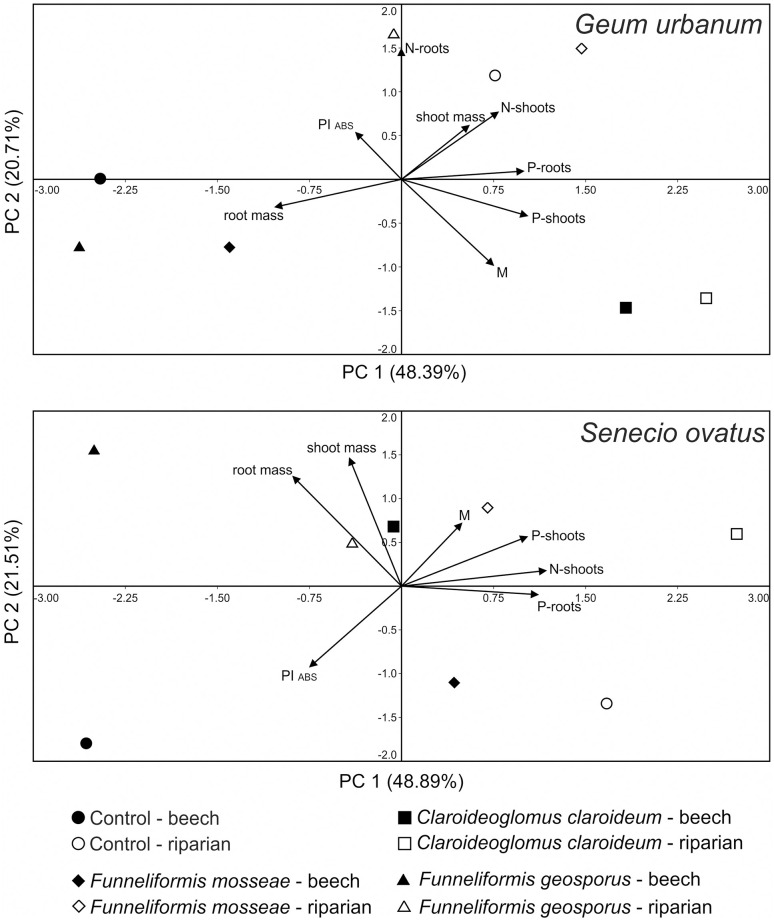
Principal component analysis ordination diagram (PC 1 vs PC 2) of *Geum urbanum* and *Senecio ovatus* parameters (M—relative mycorrhizal root length, shoot and root mass, N and P contents in shoots and roots, PI_ABS_) for samples of the two soil types and four fungal treatments. The percentage of total variance, as explained by each axis, is shown.

Principal component analysis (PCA) revealed patterns in the plant parameters across all samples (Fig 5). In the case of *G*. *urbanum*, PCA axis 1 was most influenced by root mass and P contents in shoots and roots, PCA axis 2 by N content in roots and PI_ABS_. The scatterplot showed separation of particular treatments involving the two soil types along the first axis, with the exception of *C*. *claroideum* in beech soil. Higher root mass and lower P contents in shoots and roots of plants in beech soil were most responsible for this pattern. A similar tendency was observed for *S*. *ovatus*. Plants in beech soil, which tended to be located on the left side of the diagram, were characterised by higher values of root mass and PI_ABS_, those in riparian soil by increased N content and P contents.

## Discussion

We have shown, for the first time, that the herbaceous plants *Geum urbanum* and *Senecio ovatus* growing in beech and riparian soils were slightly dependent on AMF for their performance, and that both herbs were able to grow well without the presence of AM. The direction and magnitude of the responses of both plant species to AMF were related to fungal species and soil types.

The level of AMF colonisation of *G*. *urbanum* was low; in the case of *F*. *geosporus* it was near zero. Although higher F values were noted in *S*. *ovatus* inoculated with *C*. *claroideum* and *F*. *mosseae*, the M and A parameters fluctuated around low values; similarly as in the case of *G*. *urbanum*, *F*. *geosporus* colonisation rates were negligible. The relatively low level of intensity of AMF colonisation of both species is in accordance with rates found among forest herbaceous plants in the field, though higher levels were found in some species [19]. Carrenho et al. [39] found that various AMF species colonised their hosts with different degrees of intensity. The level of colonisation was also impacted by the type of soil involved [39–42]. This may be due to the incompatibility of a particular species or strain of AMF with plant partners in a given soil type [14, 18].

Compared with control, AMF did not enhance shoot mass or photosynthetic performance index, and very few AMF effects on root mass of both plant species were noted. Moreover, no clear trends were observed regarding enhancement of N and P acquisition due to AMF. Earlier studies on the effects of inoculation with AMF on several species from Rosaceae indicated that their growth responses could be negative, neutral, or positive. On one hand, a study by Sudová and Vosátka [10] on the inoculation of *Fragaria moschata* and *Potentilla reptans* by three AMF species, including *F*. *mosseae*, showed negative growth response. However, AMF were effective in supplying P. Nevertheless, higher P content in shoots of both species were not associated with stimulation of their growth, indicating that N rather than P content is the governing factor in these specific interactions [10]. Similarly, Zobel and Moora [9] noted the absence of any growth response of *Fragaria vesca* to AMF. On the other hand, an experiment carried out by Mark and Cassells [43] on the inoculation of *F*. *vesca* with *C*. *claroideum* revealed a positive growth response. Studies on the effects of AMF on numerous species from Asteraceae showed, in most cases, positive plant response. The following results have been noted: enhanced growth of *Centaurea jacea* following inoculation with AMF soil inocula [9]; increased biomass and enhanced photosynthetic performance index of *Senecio umbrosus* following inoculation with, among others, *C*. *claroideum* and *F*. *geosporus* [44]; and enhanced biomass, but not PI_ABS_, in *Inula ensifolia*, *Rudbeckia laciniata*, and *Solidago gigantea* following inoculation with several AMF species [25, 45].

Mycorrhizal dependency of *G*. *urbanum* and *S*. *ovatus* concerning growth and N and P acquisition was near zero in both soils, but varied between AMF species in terms of elements. This indicates that neither species is dependent on AMF in this respect. This observation contradicts studies of other species from Asteraceae and Rosaceae, where the level of mycorrhizal dependency for growth was found to be high. *Osteomeles anthyllidifolia* (Rosaceae), inoculated with six AMF species simultaneously, achieved values of mycorrhizal dependency between 46% and 76%. Nevertheless, these values were related to P content in soils, being higher in soils with lower P content [46]. In the case of representatives of Asteraceae, mycorrhizal dependency of *R*. *laciniata* was 88% and 63% and for *S*. *gigantea* 90% and 82% for two soil types involved, respectively [25]. Similarly, *Bidens sandvicencis* was rated by Gemma et al. [47] as a highly AMF-dependent species, as it achieved values of mycorrhizal dependency up to 88% after being inoculated with *Rhizophagus aggregatus*. These effects were also linked with P content in soil. These studies did not focus on distinctions between the impacts of particular AMF species; however, van der Heijden et al. [37] showed that mycorrhiza-dependent plant species respond differently to different AMF species.

The effects of AMF on plant species can be multidirectional and may vary in different environmental conditions. Literature data confirm that numerous interacting factors, both abiotic and biotic, have an influence on soil microbial properties and related response of plants. For example, in natural forest ecosystems, the co-existence of trees and herbaceous species, which differ in the formation of AM, ectomycorrhiza or are non-mycorrhizal, affect the abundance of AMF propagules in soils, and thus influence the intensity of AMF colonisation degree of plants in particular sites [19, 48, 49]. Moreover, it was observed that in the restoration of degraded areas, higher AMF species richness enhanced inoculum effectiveness. The positive response from plants increased from multiple species to whole soil inoculum [50]. However, ecologists also signalise that since AMF show functional diversity and their effects are within mutualism and parasitism spectrum [51–53], the key point is to select single effective AMF species than focus on species richness [54]. No without significance in plant-AMF relationships are also other factors, like soil physical and chemical properties [46, 47, 55] as well as presence of biotic and abiotic stresses. It was reported that AMF can have positive effects on plants in the presence of stressors, such as salinity and heavy metal toxicity [56–59], by both nutritional and non-nutritional mechanisms [57, 58, 60, 61]. The formation of AM may impact on resistance of plants for pathogens and herbivores by stimulations of bacteria in soils as well as by modifications of secondary metabolite synthesis in plants [62]. Therefore, other parameters, not measured in the present study, should be examined in future in order to identify complete spectrum of *G*. *urbanum* and *S*. *ovatus* response to AMF in forest ecosystems.

In conclusion, our investigation included three AMF species and soils of two forest habitats, thus enabling us to draw strong inferences on the effect of AMF on *G*. *urbanum* and *S*. *ovatus*. We report, for the first time, that neither species is dependent on AMF for its growth and photosynthetic performance index. Moreover, we found only slight effects of AMF on N and P acquisition for both plant species, whose direction and magnitude were dependent on fungal species and soil identities. Thus, *G*. *urbanum* and *S*. *ovatus* are adapted to grow in temperate-forest soils, which can be characterised by a low level of availability of AMF propagules in comparison to other ecosystems [19, 49, 63]. This is further supported by the fact that *G*. *urbanum* is also found in habitats with disturbed soil [64] which are usually characterised by low levels of AMF abundance [65, 66].
